# COVID-19: Cultural Predictors of Gender Differences in Global Prevalence Patterns

**DOI:** 10.3389/fpubh.2020.00174

**Published:** 2020-04-30

**Authors:** Olav T. Muurlink, Andrew W. Taylor-Robinson

**Affiliations:** ^1^Sustainable Innovation, School of Business & Law, Central Queensland University, Brisbane, QLD, Australia; ^2^Infectious Diseases Research Group, School of Health, Medical & Applied Sciences, Central Queensland University, Brisbane, QLD, Australia

**Keywords:** COVID-19, dengue, transmission, culture, religion, gender, traditional clothing, facial touching

Puzzling differences are emerging between male and female infection and death rates for COVID-19 (1). We predict that this may be amplified, especially in the developing world, due to hitherto overlooked cultural factors. Currently, credible data from low- and lower middle-income countries on COVID-19 are sparse, with recorded case numbers seemingly suppressed by unreliable surveillance, lesser testing capacity and an underlying burden of infectious diseases that may mimic key symptoms, notably pyrexia. Indeed, acute undifferentiated febrile illness is a common feature of resource-limited tropical regions. Patterns of prevalence of *vector-borne* diseases in the developing world, however, offer an indication of likely COVID-19 infection and morbidity gender trends.

Cultural factors, in particular the extent to which long or “modest” clothing is worn and the convention of separating adults by gender, may inadvertently determine the rapidity and extent of the spread of communicable diseases including COVID-19. A study of six Asian countries on the prevalence of dengue showed a striking tendency toward greater infection rates for males compared to females, but only for those aged 15 or over for whom cultural differences in work patterns outside the home, social interaction and dress all apply (2). This disparity is plausibly explained as a difference in exposure to the mosquito vector and is linked to established recommendations on wearing protective clothing. However, it is noteworthy that in Brazil, where standards of modesty for male and female clothing are equivalent (3), this gender difference in dengue incidence disappears (4).

Cultures that place greater restrictions on the movement and dress of women are likely to see fewer opportunities for both vector- and air-borne pathogen transmission for women relative to men. One of the known routes of infection with SARS-CoV-2 is touching one's face, leading to public health agency advisories against this practice (5, 6). This presents a challenge to community education since this behavior is instinctive (7), habitual and very frequent (8). Yet, in conservative Muslim cultures in particular, where wearing a burka or niqab, providing full or partial coverage of the face, respectively, is relatively common in public, touching of mouth, nose and eyes by females is correspondingly restricted. Even in the increasingly observed instances of where the “modesty" function of covering the hair and face is separated from the traditional (often religious) purpose of the clothing (9), such practices have this unintended public health value. Facial covering additionally affords a limited level of filtration of air-borne droplets (10), such as those carrying virus particles. In contrast, the cultural predilection for facial hair among male Muslims is likely to further increase male exposure to the virus, particularly amongst health professionals where facial hair compromises the seal of P2/N95-standard particulate filtering respirators and surgical masks (11).

In a recent analysis of gender and COVID-19, a working group argued that “policies and health impacts have not addressed the gendered impacts of disease outbreaks” (12), but the interaction between gender roles and disease exposure was overlooked in their analysis. In other cultures, or indeed subcultures, where versions of the veil or other passive forms of discouragement of facial touching are absent, but where strict or partial segregation of genders is observed due to cultural norms (e.g., among Amish communities in the United States, or in Orthodox Jewish communities in Israel) (13, 14) pathways to community transmission are likely to be impinged. Of course, more highly-segregated workforces and family life is seen in traditional societies regardless of the prevalent religion or other belief system.

The segregation between genders is apparent even in industrialized nations, albeit less overtly, where it impacts on the involvement of women in society itself (such as the extent to which females engage in certain occupations or roles outside the home) [e.g., (15, 16)]. This lower level of engagement in society beyond the customary domestic and childcare functions may even, in extreme cases, reduce the likelihood of women attending a health clinic to receive a diagnosis (and treatment), leading to underreporting of diseases among adult females. For instance, in rural and remote regions there is often a gender imbalance in favor of male medical practitioners (17). In combination with strong cultural inhibitors that are frequently prevalent in isolated communities toward women interacting with men outside their family group (18), women may not expressly seek medical attention.

Here, we argue that cultural factors may impact on the gender balance of reported COVID-19 infection prevalence in systematic ways, particularly in conservative societies, whether religious or secular, around the world. This is to say: women may be afforded some protection by customs relating to traditional clothing; they may be placed at less risk of contracting infection through distancing from men or separation from the broader workforce and community; and — by their known reluctance to be attended by a male medical practitioner and so be less disposed to seek a qualified diagnosis — they may be underrepresented in data collected on infection and morbidity.

## Author Contributions

OM and AT-R made substantial contributions to the conception of the work and to literature search, contributed significantly to writing the manuscript, revised it critically for important intellectual content, approved its final version, and agreed to its submission.

## Conflict of Interest

The authors declare that the research was conducted in the absence of any commercial or financial relationships that could be construed as a potential conflict of interest.
